# Professional indemnity insurance rates for metabolic bariatric surgeons in Australia: survey results

**DOI:** 10.1111/ans.70087

**Published:** 2025-03-14

**Authors:** Jacob Chisholm, Lilian Kow, Adam Skidmore, Nicholas Williams

**Affiliations:** ^1^ Department of Gastrointestinal Surgery Flinders Medical Centre Adelaide Australia; ^2^ Department of Surgery Albury Wodonga Health Wodonga Australia; ^3^ School of Medicine Sydney University of Notre Dame Wagga Wagga Australia

Metabolic bariatric surgery remains the most effective, durable, and safe method of weight loss in the morbidly obese.^1^ The evidence for the cost‐effectiveness of metabolic bariatric surgery to achieve this, prevent or treat obesity‐related comorbidities, and prolong life is well established.^2^, ^3^ Metabolic bariatric surgery remains a management cornerstone for patients living with clinical obesity in this country.^4^ In Australia, most operations (97%) are performed in the private sector.^4^ Private metabolic bariatric surgeons are required to hold professional indemnity insurance (PII) to be able to practice.

To gain an understanding of PII rates for metabolic bariatric surgeons and trends around malpractice claims in Australia, a cross‐sectional online survey was developed by the Medicolegal Subcommittee and Board of the Australian and New Zealand Metabolic and Obesity Surgical Society (ANZMOSS) (Table 1). This survey was distributed via email to all Australian‐based surgical members of ANZMOSS in October 2023. A follow‐up email was sent a month later to nonrespondents.

**Table 1:** Medical indemnity insurance survey

Which state in Australia do you predominantly work from?
2 Estimated number of bariatric procedures performed in your career?
1–100
101–250
251–500
501–1000
1001–1500
Other (please specify)
3 What best describes the way you perform bariatric surgery?
Solo practitioner
Specialty group of surgeons
Solo surgeon with multidisciplinary clinic
Specialty group of surgeons and multidisciplinary
Academic/public hospital
4 Do you work primarily in a regional area?
5 Do self‐funded patients account for more than 50% of your bariatric workload?
6 How much did you pay for financial year 2022/23 in indemnity insurance?
7 How much did you pay for financial year 2023/24 in indemnity insurance?
8 Do you currently have a bariatric related claim against you?
9 How many bariatric related claims have you had against you in your career?
10 How many of these claims have you settled out of court?
11 How many claims have been dropped without finding you at fault?
12 Have cases against you gone to trial?
13 How many times has a court found for the plaintiff in a case where professional negligence has been alleged against you?
14 How many times has a court found in your favour where professional negligence has been alleged against you?
15 Has your current insurer forced you to change how you deliver bariatric care?
16 Have you been a defendant in a claim where the expert on the standard of care was not a currently practising bariatric surgeon?
17 Are you aware of the Premium Support Scheme to assist with the costs of indemnity insurance?
18 If the PSS kicks in when your indemnity insurance is more than 7.5% of your gross private medical income would you qualify?
19 Are you considering ceasing bariatric surgery due to the financial burden of indemnity insurance?
20 Have you already ceased performing bariatric surgery due to the cost of indemnity insurance?

The response rate to the survey was 52% (115/222). The mean reported annual cost of PII in 2022/23 was $51,748 ± $34,687 (range $4000–$230,000). The mean annual cost of PII in 2023/24 was $69,933 ± $60,181 (range $5500–$500,000). This represented an annual increase of 35%.

There was a total of 142 claims. Forty‐one percent (47/115) of surgeons reported at least one metabolic bariatric‐related malpractice claim in their career (range 1–20). Only 3/142 (2%) of claims came to trial. There were no documented cases where a court found for the plaintiff when professional negligence had been alleged against the surgeon. Seventy‐two percent (34/47) of surgeons involved in claims were defendants where the expert on the standard of care was not a currently practicing metabolic bariatric surgeon.

The premium support scheme (PSS) provides a government subsidy to assist doctors with the cost of their PII. To be eligible for PSS, a doctor must have gross indemnity costs that are more than 7.5% of their gross private medical income. Sixty‐two percent (71/115) of respondents were aware of the PSS to assist with the cost of PII. Forty‐one percent (47/115) qualified for the PSS, as their indemnity insurance was more than 7.5% of gross private medical income.

Ten percent (11/115) of respondent surgeons' insurers forced a change to how they delivered metabolic bariatric care. Three out of one hundred and fifteen (3%) respondents had already ceased performing metabolic bariatric surgery due to the cost of PII. Thirty‐seven percent (43/115) were considering ceasing metabolic bariatric surgery due to the financial burden of PII.

Variation in PII was seen across different states (Table 2). The greatest increase was seen in Victoria. PII rates increased with the total volume of cases performed (Table 2). This is perhaps reflective of increasing income with PII rates based on reported income to the medical defence organizations (MDO). It may also suggest increased claims. More malpractice claims are made against surgeons who have performed the greatest number of cases.^5^

**Table 2:** State, volume, practice type

		*n*	Mean PII 22/23	Mean PII 23/24	Change
State	New South Wales	27 (23%)	$54 844 ± $24 220	$72 165 ± $40 907	↑32%
	Victoria	34 (30%)	$49 574 ± $41 744	$77 152 ± $86 851	↑56%
	Queensland	29 (25%)	$55 488 ± $33 732	$70 940 ± $52 928	↑28%
	Western Australia	11 (7%)	$62 085 ± $48 192	$68 295 ± $45 513	↑10%
	South Australia	12 (10%)	$31 553 ± $16 521	$39 429 ± $14 325	↑25%
Total volume cases	1–100	20 (17%)	$29 869 ± $16 562	$42 783 ± $21 156	↑43%
	101–250	22 (19%)	$44 386 ± $19 609	$54 955 ± $26 336	↑24%
	251–500	24 (21%)	$43 231 ± $26 872	$55 195 ± $31 956	↑28%
	501–1000	13 (11%)	$69 009 ± $44 701	$97 795 ± $79 744	↑42%
	1001–1500	11 (10%)	$50 464 ± $21 639	$64 938 ± $21 558	↑29%
	>1500	27 (23%)	$72 236 ± $45 144	$102 604 ± $92 402	↑42%
Practice type	Solo practitioner	34 (30%)	$54 851 ± $40 430	$77 719 ± $85 101	↑42%
	Specialty group surgeons	6 (5%)	$33 586 ± $11 700	$41 206 ± $14 492	↑23%
	Solo surgeon with MDT	36 (31%)	$57 914 ± $30 467	$78 217 ± $54 307	↑35%
	Specialty group surgeons and MDT	40 (35%)	$45 980 ± $35 045	$59 706 ± $40 369	↑30%
	Academic/public hospital	16 (14%)	$33 427 ± $25 130	$39 074 ± $27 921	↑17%

Inexperienced surgeons who are just starting out in their metabolic bariatric career have been subjected to a 43% increase with a history of claims unlikely (Table 2). This significant upfront financial burden will act as a disincentive for surgeons wanting to enter the subspecialty of metabolic bariatric surgery.

Rates of PII are influenced by the way metabolic bariatric surgeons practice (Table 2). PII rates are increased when the metabolic bariatric surgeon is working solo, with or without a multidisciplinary team. This implies that MDOs are taking into consideration how surgeons manage their clinic when determining levels of PII.

The prevalence of clinically severe obesity is greater in the rural community in Australia^6^ and as a result, rural metabolic bariatric surgical services are invaluable. The rural metabolic bariatric surgeon is subject to a greater amount of PII and has seen a greater increase in the last 12 months compared with city colleagues (Table 3). The outcome will be closure of existing clinics, leading to further access issues for those patients most in need.

**Table 3:** Rural clinic, self‐funded, claims history

		*n*	Mean PII 22/23	Mean PII 23/24	Change
Rural	No	74 (64%)	$48 270 ± $32 586	$63 290 ± $44 724	↑29%
	Yes	41 (36%)	$56 854 ± $38 569	$81 922 ± $80 266	↑44%
>50% self‐funded	No	71 (62%)	$48 270 ± $30 546	$57 698 ± $30 569	↑20%
	Yes	44 (36%)	$57 360 ± $40 580	$89 676 ± $86 224	↑56%
Claims	0	68 (59%)	$37 917 ± $18 261	$49 012 ± $21 019	↑29%
	1–3	35 (30%)	$66 405 ± $41 084	$82 808 ± $83 525	↑25%
	4–20	12 (10%)	$87 375 ± $45 608	$116 167 ± $79 287	↑33%

Most metabolic bariatric patients in Australia have their surgeries utilizing private health insurance to assist with payment. There are patients, however, who will elect to self‐fund without private health insurance for their surgery. They will utilize their personal funds or access their superannuation (compulsory savings). This is an expensive way to obtain surgery, and not all metabolic bariatric surgeons offer this as an option. Premiums paid by surgeons where self‐funded patients form a significant part of their workload suggest an increased risk of claims in this group (Table 3). This may relate to the amount of money the patient has paid for their surgery with no insurance cover. If the patient suffers a less than expected outcome, they are more likely to make a claim due to the inflated amount of money they paid for their surgery.

The amount of PII paid appears to have a direct relationship with the total number of claims experienced (Table 3). It also, however, showed a significant increase for surgeons who have no history of claims. Metabolic bariatric surgeons are being penalized collectively under the assumption that all will be subject to claims in the future when it may not be the case.

The use of nonexpert witnesses in metabolic bariatric claims is now common in Australia. There is evidence that the use of such witnesses is associated with the occurrence of a claim but not a payout.^5^ In response, ANZMOSS has established the Independent Medicolegal Advisory Panel (iMAP) to provide independent medical opinion by experts in the field of metabolic bariatric surgery. Expert witnesses on this panel are selected based on criteria established by the American Society of Metabolic and Bariatric Surgery (ASMBS) with impartiality and medical accuracy paramount.^7^

MDOs have reported a doubling in civil claims frequency for metabolic bariatric surgeons with upward pressure on premiums as a result.^8^ We believe the reason for this increase is the ‘no win, no fee’ legal approach. This system has merits as it enables patients with reduced means to access legal redress if needed. It does also however encourage the pursuit of claims that are often frivolous and without merit. The tendency for MDOs to settle rather than defend unwarranted claims leads to further claims and on it continues.

Potential solutions include state‐based tort reform with caps on damages and contingency fees. A closed claims registry should be established with contributions from all MDOs. This will give us a better understanding of the causes of malpractice claims, with subsequent quality improvement possible. We also need to discredit ‘nonexpert’ witnesses in claims cases, and iMAP is an attempt to make that change.

This survey has demonstrated unsustainable PII rates. Metabolic bariatric surgeons will close their clinics as a result (there is concern for other specialties such as spinal surgery and obstetrics) and access to this life‐saving and cost‐effective treatment will be even further limited.

## Author contributions

**Jacob Chisholm:** Conceptualization; formal analysis; writing – original draft; writing – review and editing. **Lilian Kow:** Writing – original draft; writing – review and editing. **Adam Skidmore:** Conceptualization; writing – original draft; writing – review and editing. **Nicholas Williams:** Conceptualization; writing – original draft; writing – review and editing.
